# miR-26 Induces Apoptosis and Inhibits Autophagy in Non-small Cell Lung Cancer Cells by Suppressing TGF-β1-JNK Signaling Pathway

**DOI:** 10.3389/fphar.2018.01509

**Published:** 2019-01-09

**Authors:** Yi He, Hao Liu, Lianyong Jiang, Bi Rui, Ju Mei, Haibo Xiao

**Affiliations:** Department of Cardiothoracic Surgery, School of Medicine, Xinhua Hospital, Shanghai Jiao Tong University, Shanghai, China

**Keywords:** NSCLC, miR-26, TGF-β, JNK, apoptosis, autophagy

## Abstract

Non-small cell lung cancer (NSCLC) is one of the causes of cancer mortality worldwide. The role of miR-26 in the development and progression of NSCLC remains largely unknown. In this study we found an abnormal expression of miR-26 in human NSCLC tissues. It was found that miR-26 mimics induced cell apoptosis and promoted caspase-3, 9 activities in human NSCLC cells. The miR-26 inhibitor enhanced the expression of the light chain 3 (LC3) protein and the autophagy related genes in NSCLC cells. Moreover, miR-26 regulated apoptosis and autophagy by inhibiting TGF-β expression in a JNK dependent manner. In addition, miR-26 mimics induced cell apoptosis, was involved in the endoplasmic reticulum stress (ERS) signaling pathway. Down-regulation of the ERS, inhibited apoptosis which was induced by miR-26 mimics in NSCLC cells. In *in vivo* studies, TUNEL staining revealed that the number of TUNEL positive cells of the tumor tissue in the miR-26 treatment group, were significantly increased in comparison with the control group, while the number of TUNEL positive cells in the tumor tissue were remarkably decreased in the groups treated with miR-26, combined with the TGF-β1 inhibitor or JNK inhibitor. Additionally, the immunoreactivity of TGF-β1 in the cells treated with the miR-26 inhibitor, decreased in comparison to the control group. Our results indicated that miR-26 induced apoptosis and inhibited autophagy in human NSCLC cells through the TGF-β1-JNK signaling pathway, suggesting that miR-26 could be a potential novel target for the treatment of NSCLC.

## Introduction

Lung cancer is the leading cause of cancer mortality in China (Hong et al., 2015; Zhang et al., 2017). Non-small cell lung cancer (NSCLC) accounts for nearly 85% of all types of lung cancers (Hutchinson, 2017). Currently, surgical resection is an effective treatment for NSCLC and can promotes a 5-year survival rate for NSCLC patients (Wu et al., 2017). However, because of distant metastasis as well as a shortage of effective chemotherapeutics, there is only a 10–15% 5-year survival rate for stage IIIA NSCLC (Ripley et al., 2016; Palka et al., 2017). Therefore, understanding the underlying mechanisms of the progression of NSCLC, as well as novel therapeutic strategies, is critical in order to improve patients’ survival time.

MicroRNA (miRNA) is a class of small non-coding RNAs, which regulates gene expression by binding to mRNA (Williams et al., 2016). It is well known that miRNA participates in numerous biological processes of various human diseases, including cancer (Jing et al., 2015; Singh and Sen, 2017). miR-26, a functional miRNA, has been investigated in various human cancers (Kwon et al., 2015; Deng et al., 2017; Jin et al., 2017; Li et al., 2017). A previous study reported that the expression of miR-26 was down-regulated in bladder cancer (Lin et al., 2013). In addition, miR-26 was found to be down-regulated in breast cancer tissues and cell lines. Up-regulation of miR-26 expression mediated apoptosis through endogenous and exogenous pathways by directly binding to the 3′-UTR of MTDH and EZH2 (Zhang et al., 2011). However, the expression and effect of miR-26 in NSCLC is still obscure. A transforming growth factor (TGF-β), a multifunctional cytokine, can induce cell apoptosis and autophagy in various human diseases (Jiang et al., 2016). A previous study showed that the TGF-β induced autophagy and apoptosis by regulating the expression of Disabled-2 (Xu et al., 2012). JNK, a protein kinase of the MAPK family, plays a critical role in the biological process of the apoptosis and autophagy of cancer cells (Díaz et al., 2017; Lu et al., 2017). A recent study has demonstrated that the TGF-β induced autophagy and apoptosis in hepatocellular carcinoma and mammary carcinoma cells, through mediating the JNK pathways (Kiyono et al., 2009). However, the role of the TGF-β on NSCLC and whether there is a crosstalk between the TGF-β and JNK in NSCLC, remains unknown. ERS, a fundamental property of all cells, is critical in regulating cell growth and apoptosis (Liu et al., 2017; Song et al., 2017). A previous study revealed that ERS induced cell apoptosis, by arresting cells at the G1 phase (Thinon et al., 2016). Additionally, ERS could re-establish cellular homeostasis by serving as a checkpoint molecule (Verfaillie et al., 2013). ER stress-inducing agents exhibited increased cell apoptosis in Perk^-/-^ mouse embryonic fibroblasts (Gupta et al., 2012).

In this study, we explored the role and molecular mechanism of miR-26 on the development and progression of NSCLC. We examined how miR-26 mimics or inhibitors, regulate cell apoptosis and autophagy, and related signaling pathways in NSCLC both *in vitro* and *in vivo*. We found that miR-26 induced cell apoptosis and inhibited autophagy by targeting the TGF-β expression in a JNK dependent manner in human NSCLC cells. Moreover, miR-26 regulated cell apoptosis was involved in ER stress in human NSCLC cells. Down-regulation of ERS inhibited cell apoptosis, regulated by miR-26 mimics. Our results provided a potential therapeutic strategy for improving NSCLC treatment.

## Materials and Methods

### Patients and Tissue Samples

This study was approved by the Human Research Ethics Committee of Xin Hua Hospital (NO: 2015-035). The NSCLC tissues and adjacent non-tumor lung tissues were obtained from six patients who underwent the primary surgical resection of NSCLC at the Xin Hua Hospital (Shanghai). All participants provided written informed consent. The samples contained well-documented clinicopathological information, including age, gender, tumor size and location, tumor differentiation, invasion depth, lymph node metastasis, distant metastasis, tumor stage, and follow-up data. Tissues were immediately frozen in liquid nitrogen after resection and stored at -80°C. Both NSCLC tissues and the adjacent non-tumor lung tissues were confirmed by a pathological examination.

### Regents

The synthetic miR-26 mimics, miR-26 inhibitor oligonucleotides, as well as the control inhibitor oligonucleotides, were purchased from Sangon Biotech (Shanghai, China). The TGF-β1 inhibitor was purchased from Sigma (St. Louis, MO, United States). The TGF-β1 was obtained from R&D Systems (Minneapolis, MN, United States). The JNK inhibitor and Lipofectamine 2000, were purchased from Invitrogen (Carlsbad, CA, United States). The Apoptosis Detection Kit was acquired from BioLegend (San Diego, CA, United States).

### Cell Culture

The NSCLC cell lines A549, H1703, and 801D were purchased from the Shanghai Institute of Chinese Academy of Sciences (Shanghai, China). All cells were maintained in RPMI-1640 and supplemented with a 10% inactivated fetal bovine serum (Gibco, Grand Island, NY, United States), 100 U/mL penicillin and 100 μg/mL streptomycin (Gibco) in a humid environment at 37°C with 95% air and 5% CO_2_.

### Plasmid Construction and Transfection

The sequence of the JNK was designed to be amplified and cloned into a pCDNA3.1 expression vector (Invitrogen). Transfection was performed using the Lipofectamine 2000 reagent. Briefly, cells were inoculated into 6-well plates and a plasmid and liposomal transfection reagent was added to the cells.

### Lentivirus-Mediated siRNA Knockdown

The lentiviral expression systems were purchased from System Biosciences (SBI, Mountain View, CA, United States). Oligonucleotides of siRNA for Chop, ATF-4, Bip, XBP-1, and the control were obtained from Sangon Biotech (Shanghai). After co-transfection, the virus media was harvested. Cells were infected for 72 h with a lentivirus containing Chop, ATF-4, Bip, XBP-1, and control siRNA.

### Caspase Activity

The activation of caspase-3, 9 was detected with a caspase activity assay. Briefly, cells in 96-well plates were treated with the miR-26 inhibitor or the control inhibitor. After incubation for 24 h, 20 μL of lysis buffer was added to each well. The cell lysate was incubated with 5 μL of a chromogenic substrate at room temperature in the dark for 20 min. The results were measured with a plate reader at 560 nm light length.

### Quantitative Real-Time PCR

A TRIzol reagent (Invitrogen, Carlsbad, CA, United States) was used to extract the total RNA. The complementary DNA samples were subjected to denaturing at 95°C for 10 s, annealing at 55°C for 15 s, and extension at 72°C for 30 s, for 45 cycles using a High Capacity cDNA Reverse Transcription Kit (Applied Biosystems Inc.). The following primer pairs were used (Table 1): Chop, ATF-4, Bip, XBP-1, DR5, BECLIN1, ATG5, ATG7, DAPK, and GAPDH. Relative gene expressions were quantified by real-time PCR, using the SYBR Premix Ex Taq^TM^ II (TaKaRa Bio, Dalian, China) on a Lightcycler 480 RealTime PCR System (Roche Diagnostics, Meylan, France).

**Table 1:** Primers used for PCR amplification.

CHOP	Forward primer, 5′-GAACCTGAGGAGAAGAGTGTTCCA-3′
	Reverse primer, 5′-AGTGACTCAGCTGCCATCTCTGT-3′
ATF-4	Forward primer, 5′-CTGGAGAGAAGATGGTAGCAGCAA -3′
	Reverse primer, 5′-GCCCTCTTCTTCTGGCGGTA-3′
Bip	Forward primer, 5′-CCAACTGTTACAATCAAGGTC-3′
	Reverse primer, 5′-ACGAGGAGCAGGAGGAAT-3′
XBP-1	Forward primer, 5′-TGCTGAGTCCGCAGCAGGTG-3′
	Reverse primer, 5′-GCTGGCAGGCTCTGGGGAAG-3′
DR5	Forward primer, 5′-TCAAAGGACACGGCAGAGCCTGTGCCA-3′
	Reverse primer, 5′-GGGAGCCGCTCATGAGGAAGTTGG-3′
BECLIN1	Forward primer, 5′-ACCGTGTCACCATCCAGGAA-3′
	Reverse primer, 5′-GAAGCTGTTGGCACTTTCTGT-3′
ATG5	Forward primer, 5′-AGCAACTCTGGATGGGATTG-3′
	Reverse primer, 5′-CACTGCAGAGGTGTTTCCAA-3′
ATG7	Forward primer, 5′-ACCCAGAAGAAGCTGAACGA-3′
	Reverse primer, 5′-AGACAGAGGGCAGGATAGCA-3′
DAPK	Forward primer, 5′-TCTACCAGCCACGGGACTTC-3′
	Reverse primer, 5′-GCTGGCCTGTGAGTAGACGT-3′
GAPDH	Forward primer, 5′-TGGAAGGACTCATGACCACA-3′
	Reverse primer, 5′-TTCAGCTCAGGGATGACCTT-3′


### Western Blotting

Protein was resolved by an SDS-PAGE. Subsequently, gel-separated proteins were blotted. The membranes were probed with primary antibodies LC3, TGF-β1 (Abcam, Cambridge, MA, United States) and Bcl, Bax, BECLIN1, ATG5, ATG7, DAPK, JNK, Chop, ATF-4, Bip, and XBP-1 antibodies (Cell Signaling Technology, Beverly, MA, United States) were diluted according to the manufacturer’s instructions. The membranes were then probed with horseradish peroxidase-conjugating (HRP) secondary antibody (1:10000; GE Healthcare, Tokyo, Japan). The proteins in the blots were visualized using the ECL plus system (Amersham Pharmacia Biotech, Buckinghamshire, United Kingdom) to capture the images.

### *In situ* Hybridization (ISH) Staining

The slides were cut from paraffin-embedded tissue to evaluate the miRNA-26 expression by ISH. In brief, the slides were incubated at 60°C for 1 h, deparaffinized in xylene, and rehydrated with graded alcohol washes. Slides were washed and digested, then hybridized at 55°C for 2 h with 50 nmol/L locked nucleic acid -modified digoxigenin-labeled probes for miRNA-26 (Boster, Wuhan, China). Slides were placed in a blocking solution for 1 h at room temperature. An antibody signal was detected with a 4-nitro-blue tetrazolium and 5-bromo-4-chloro-3′-indolylphosphate substrate (Roche, Mannheim, Germany).

### Flow Cytometry

To detect cell apoptosis, transfected or treated cells were double stained with an annexin V-FITC/7-amino-actinomycin D (7-AAD) kit (Beckman Coulter) according to the manufacturer’s protocol. The stained cells were immediately analyzed by flow cytometry on the FACS calibur (BD Biosciences, CA, United States).

### Cell Cycle Analysis

The cell cycle was assessed using the GENMED Universal periodic flow cytometry kit (Genmed Scientifics Inc., United States). Cells were seeded in 6-well plates and incubated with the miR-26 mimics at 37°C for 48 h in a humidified chamber containing 5% CO_2_.

### Luciferase Reporter Assays

The promoter of the TGF-β1 was amplified and cloned into a pGL 3.0 luciferase reporter plasmid. Cells were then transfected with the pRL-CMV renilla luciferase reporter and the pGL 3.0 luciferase reporter plasmid. The activities of the luciferases were detected using a dual luciferase reporter assay system (Promega).

### Xenograft Nude Mouse Model

The Specific-pathogen-free (SPF)-grade nude mice (4–6 weeks of age) were obtained from the Model Animal Research Center of Nanjing University (Nanjing, Jiangsu, China), and housed with a pathogen-free fodder, equipment, and environment. The control, miR-26 inhibitor, miR-26 inhibitor + TGF-β1 inhibitor, miR-26 inhibitor + JNK inhibitor treated A549 cells were subcutaneously injected at the inguinal region of the nude mice, in a SPF-grade ultraclean work station. Using the vernier calipers, tumor diameters were measured every 2 days after 2 weeks to calculate the tumor volume: TV (mm^3^) = d2 × D/2, where d and D represent the shortest and the longest diameters, respectively. The mice were sacrificed 30 days after the cell implantation, and the tumors were extracted.

### Histopathological Analyses

Lungs cancer tissues were obtained from the sacrificed mice. The tissues were embedded in paraffin and sets of different consecutive 5-um-thick sections were acquired using an automatic microtome (SLEE Medical GmbH, Germany). The set of slides were processed for immunohistochemical staining using an anti-TGF-β1 antibody (1:100, Abcam).

### TUNEL Staining

After the mice were sacrificed, the lung cancer tissues were embedded, sectioned, and deparaffinized. The sections were incubated with proteinase K for 1 h at room temperature. Sections were then treated with 2% H_2_O_2_ in distilled water for 30 min at room temperature. After the enzymatic reaction, sections were washed with PBS and incubated with anti-digoxigenin peroxidase conjugate for 30 min at room temperature in a humidified chamber. Sections were stained with diaminobenzine and counterstained with hematoxylin and observed under a light microscope.

### Statistical Analysis

The data were analyzed using the SPSS 17.0 software (SPSS Inc., Chicago, IL, United States). The comparison between the two groups was analyzed by an unpaired Student’s *t*-test and multiple comparisons were compared by a one-way ANOVA analysis of variance followed by a Dunnett’s test. Statistical significance was defined as *P* < 0.05.

## Results

### miR-26 Induced Apoptosis in NSCLC Cells

**FIGURE 1:**
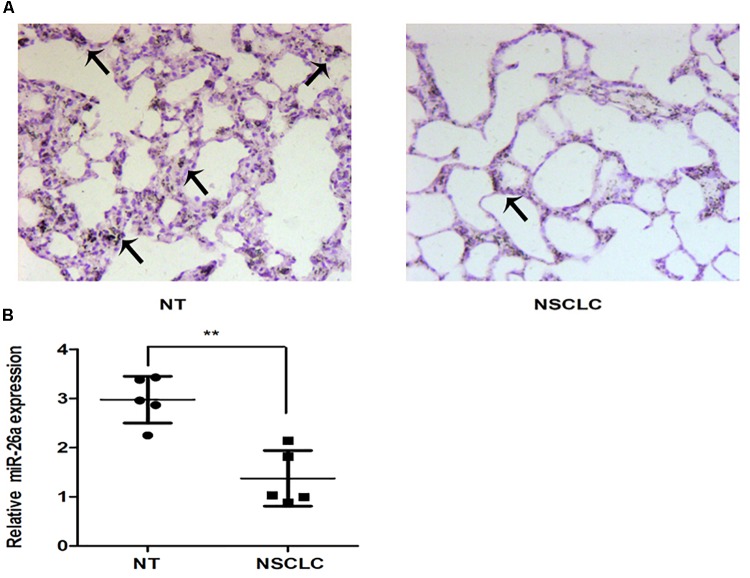
The expression of miR-26 in non-small cell lung cancer. **(A)** The representative results of ISH staining in normal tissues and lung cancer tissues. **(B)** The miR-26 expression was detected in normal tissues and lung cancer tissues. Arrows represented the positive cells.

**FIGURE 2:**
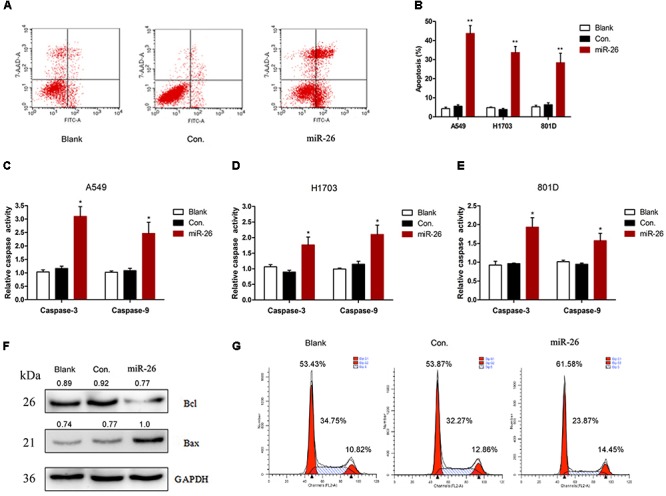
miR-26 induced cell apoptosis in non-small cell lung cancer cells. **(A,B)** Flow cytometry showed that miR-26 increased cell apoptosis in A549 cells. **(C–E)** Caspase activity assay showed increased Caspase-3 and caspase-9 activities after treatment with miR-26 in A549 cells. **(F)** Western blot showed a decreased expression of the Bcl protein and an increased expression of the Bax protein following miR-26 treatment in A549 cells. **(G)** miR-26 caused cell cycle arrest in A549 cells. ^∗^*P* < 0.05 vs. Blank, ^∗∗^*P* < 0.01 vs. Blank.

The *in situ* hybridization of miR-26 in adjacent non-tumor lung or NSCLC tissues was performed and the representative result is shown in Figures 1A,B. The expression level of miR-26 in NSCLC patients was relatively lower than the expression in adjacent non-tumor lung tissues (Figure 1C). In order to examine the effect of miR-26 on apoptosis in NSCLC cells, flow cytometry was performed in A549 cells after treatment with miR-26 mimics at a final concentration of 20 nM. In comparison with the non-treatment counterparts, miR-26 mimics treatment significantly increased the number of apoptotic cells (Figures 2A,B). Furthermore, we examined the activities of caspase-3 and caspase-9 in A549 cells. The results revealed that miR-26 mimics treatment significantly increased the activities of caspase-3 and caspase-9 in A549 cells, compared with the non-treatment counterparts (Figures 2C–E). The expression apoptosis related proteins was also examined and the results showed that Bcl was obviously decreased, while Bax was obviously increased following miR-26 mimics treatment in A549 cells (Figure 2F). In addition, to examine whether miR-26 mimics induces cell cycle arrest, we performed a flow cytometry analysis to investigate the cell cycle distribution after miR-26 mimics treatment. The result showed that the proportion of the cell cycle arrest in the G0/G1 phase and G2/M phase, obviously increased when treated with miR-26 mimics (Figure 2G). These results suggest that miR-26 induced apoptosis and the cell cycle arrest in NSCLC cells.

### miR-26 Down-Regulation Induced Autophagy in NSCLC Cells

**FIGURE 3:**
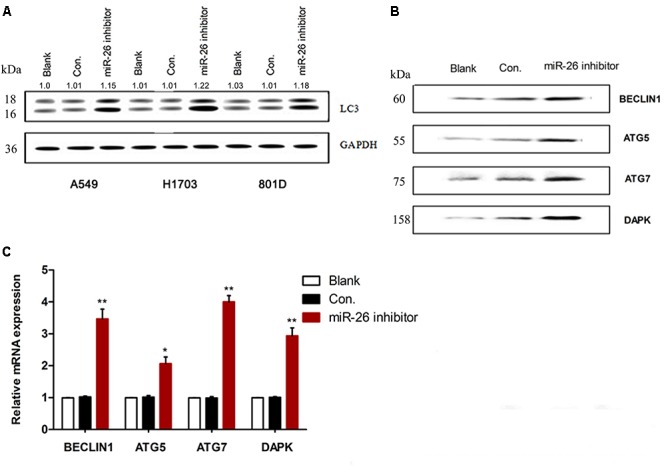
miR-26 inhibitor induced autophagy in non-small cell lung cancer cells. **(A)** Western blotting showed an increased expression of the LC3 protein following miR-26 inhibitor treatment in NSCLC cells. **(B,C)** Western blot and RT-PCR showed an increased expression of autophagy related protein molecules and mRNA of BECLIN1, ATG5, ATG7, and DAPK following miR-26 inhibitor treatment in A549 cells. ^∗^*P* < 0.05 vs. Blank, ^∗∗^*P* < 0.01 vs. Blank.

To understand the effect of miR-26 on the autophagy of NSCLC cells, we examined the expression of autophagy related molecules using Western blotting and RT-PCR in A549 cells. In comparison with the non-treatment counterparts, 50 nM of miR-26 inhibitor treatment significantly increased the protein expression of LC3 (Figure 3A). In addition, miR-26 inhibitor treatment also increased the protein expression of BECLIN1, ATG5, ATG7, and DAPK compared with the non-treatment counterparts (Figure 3B). Moreover, the mRNA expression of BECLIN1, ATG5, ATG7, and DAPK increased in the miR-26 inhibitor treatment group compared with the non-treatment counterparts in A549 cells (Figure 3C).

### TGF-β1 Was a Direct Target of miR-26 in A549 Cells

**FIGURE 4:**
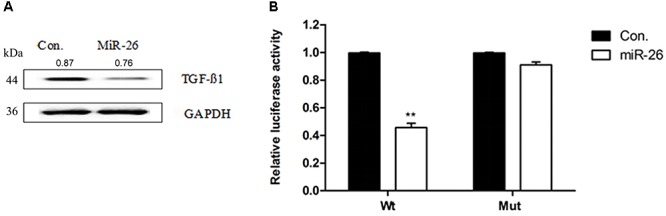
miR-26 inhibited TGF-β1 in non-small cell lung cancer cells. **(A)** Western blotting showed a decreased expression of the TGF-β1 protein following miR-26 treatment in A549 cells. **(B)** luciferase reporter assay revealed increased ASPP2 3’UTR luciferase activity in mutant A549 cells. ^∗∗^*P* < 0.01 VS Con.

To verify whether miR-26 directly targets TGF-β1, we constructed luciferase-reporter plasmids containing the wt or mutant 3′-UTR segments of TGF-β1. The wt or mutant reporter plasmid was co-transfected into A549 cells along with the miR-26 or control. miR-26 significantly decreased the relative luciferase activity when co-transfected with the wt reporter plasmid. However, the mutant reporter plasmid reversed the miR-26 mediated decrease in luciferase activity (Figure 4B). The protein expression of TGF-β1 was significantly decreased following miR-26 treatment in A549 cells (Figure 4A), consistently. These findings suggest that miR-26 suppressed TGF-β1 by directly binding to the 3′-UTR of TGF-β1.

### miR-26 Down-Regulation Regulated the TGF-β1 Signaling Pathway in a JNK-Dependent Manner in A549 Cells

**FIGURE 5:**
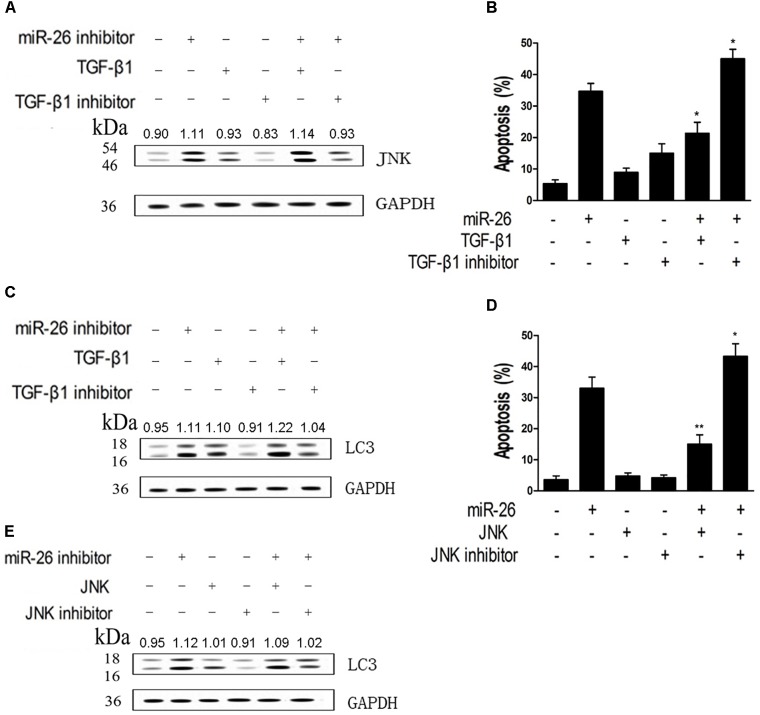
miR-26 inhibitor induced autophagy and apoptosis by targeting TGF-β1 in a JNK-dependent manner. **(A)** Western blot on the protein expression of JNK following treatment with the miR-26 inhibitor combined with the TGF-β1 or TGF-β1 inhibitor in A549 cells. **(B)** Flow cytometry on cell apoptosis in A549 cells treated with miR-26 followed by treatment with the TGF-β1 or TGF-β1 inhibitor. **(C)** Western blot on the protein expression of LC3 following treatment with the iR-26 inhibitor combined with the TGF-β1 or TGF-β1 inhibitor in A549 cells. **(D)** Flow cytometry on cell apoptosis in A549 cells treated with the miR-26 and the JNK or JNK inhibitor. **(E)** Western blot on the protein expression of LC3 following treatment with the miR-26 inhibitor combined with the JNK or JNK inhibitor in A549 cells. ^∗^*P* < 0.05 VS miR-26 group, ^∗∗^*P* < 0.01 VS miR-26 group.

We assessed whether down-regulation of miR-26 affected JNK protein expression in A549 cells. Cells were treated with the miR-26 inhibitor, the TGF-β1 or TGF-β1 inhibitor. The increased JNK protein expression was detected in the miR-26 inhibitor treatment, which was further enhanced with TGF-β1 transfection. However, the TGF-β1 inhibitor notably reversed the increase of JNK protein expression induced by the miR-26 inhibitor (Figure 5A). We further tested the apoptosis in cells treated by miR-26 mimics and TGF-β1, JNK, or TGF-β1 inhibitor and JNK inhibitor. The results revealed that TGF-β1 or JNK treatment significantly decreased cell apoptosis induced by miR-26 mimics. Whereas, TGF-β1 inhibitor or the JNK inhibitor combined with miR-26 mimics treatment significantly increased apoptosis of A549 cells compared with the miR-26 mimics treatment alone (Figures 5B,D). Moreover, the protein expression of LC3 was notably increased in the TGF-β1 or JNK combined miR-26 inhibitor, while decreased in the TGF-β1 inhibitor or JNK inhibitor combined with the miR-26 inhibitor, compared with the miR-26 inhibitor treatment alone (Figures 5C,E).

### miR-26 Regulated Autophagy and Apoptosis Were Related to ERS Signaling

**FIGURE 6:**
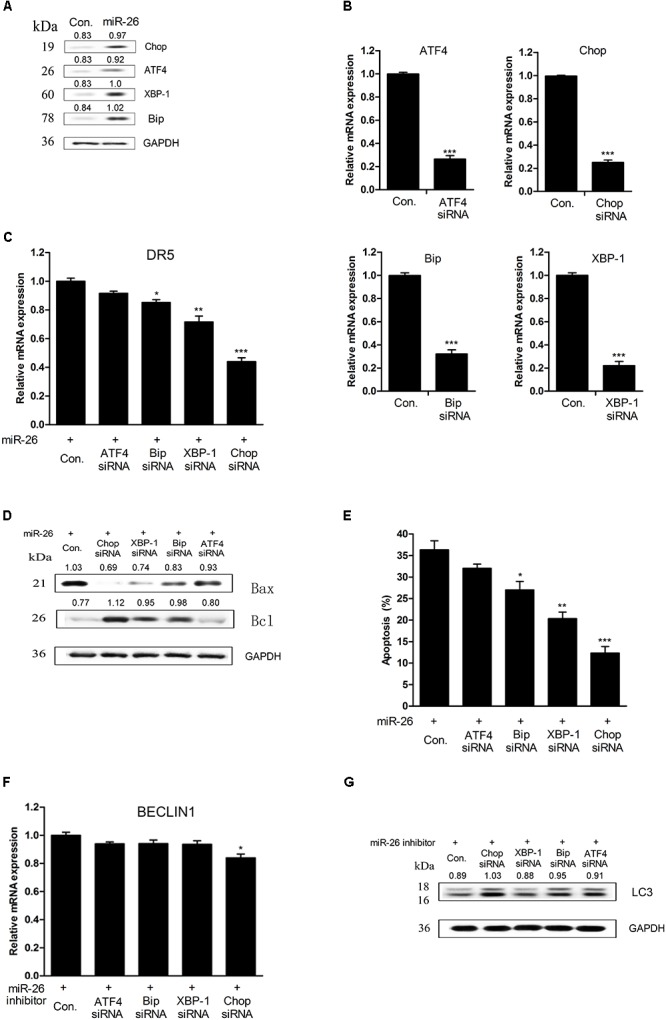
miR-26 regulated autophagy and apoptosis were correlated with ERS signaling. **(A)** Western blot on the protein expression of Chop, ATF-4, Bip, and XBP-1 following miR-26 treatment in A549 cells. **(B)** RT-PCR on the mRNA expression of Chop, ATF-4, Bip, and XBP-1 following treatment with a siRNA of Chop, ATF-4, Bip, and XBP-1. **(C)** The mRNA expression of DR5 following a siRNA of Chop, ATF-4, Bip, and XBP-1 combined with the miR-26 treatment. **(D)** Western blot on the protein expression of Bcl and Bax following a siRNA of Chop, ATF-4, Bip, and XBP-1 combined with the miR-26 treatment. **(E)** Flow cytometry on cell apoptosis detected following a siRNA of Chop, ATF-4, Bip and XBP-1 combined with miR-26 treatment. **(F)** RT-PCR on the mRNA expression of BECLIN detected following a siRNA of Chop, ATF-4, Bip, and XBP-1 combined with miR-26 inhibitor treatment. **(G)** Western blot on the protein expression of LC3 detected following a siRNA of Chop, ATF-4, Bip, and XBP-1 combined with the miR-26 inhibitor treatment. ^∗^*P* < 0.05 VS Con., ^∗∗^*P* < 0.01 VS Con., ^∗∗∗^*P* < 0.001 VS Con.

We examined whether ERS signaling was involved in miR-26 regulated autophagy and apoptosis in NSCLC cells. It was found that the protein expression of Chop, ATF-4, Bip, and XBP-1 were up-regulated after treatment with miR-26 mimics (Figure 6A). We down-regulated Chop, ATF-4, Bip, and XBP-1 with siRNA transfection and the efficiency of the transfection was confirmed by a real-time PCR (Figure 6B). Furthermore, the apoptosis in cells treated with miR-26 mimics and Chop, ATF-4, Bip, and XBP-1 siRNA was detected. The results showed that miR-26 mimics combined with Bip, XBP-1, and Chop siRNA significantly decreased apoptosis of A549 cells (Figure 6E). The mRNA expression of DR5 was significantly decreased following siRNA of Chop, Bip, and XBP-1 combined with the miR-26 mimics treatment (Figure 6C). Consistently, the protein expression of Bcl was notably increased, while the Bax expression decreased following siRNA of Chop, Bip, and XBP-1 combined with the miR-26 mimics treatment (Figure 6D). However, the protein expression of LC3 was not obviously changed following siRNA of Bip, ATF-4, and XBP-1 combined with the miR-26 inhibitor treatment, except for the Chop siRNA treatment (Figure 6G). In addition, the mRNA expression of BECLIN1 did not change following siRNA of Bip, ATF-4, and XBP-1 combined with the miR-26 inhibitor treatment except for the Chop siRNA treatment (Figure 6F).

### miR-26 Inhibited NSCLC Growth *in vivo*

**FIGURE 7:**
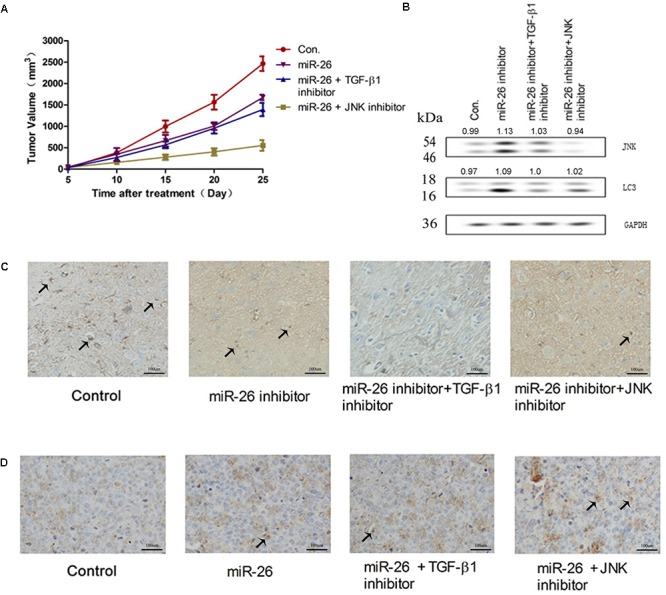
miR-26 inhibited NSCLC growth *in vivo*. **(A)** The tumor volume of mouse xenografts treated with the miR-26 and/or the TGF-β1 inhibitor or JNK inhibitor. **(B)** The protein expression of JNK and LC3 in tumors treated with the miR-26 inhibitor and/or the TGF-β1 inhibitor or JNK inhibitor. **(C)** Immunohistochemistry of TGF-β1 in the lung cancer tissues with different treatments. **(D)** TUNEL staining on cell apoptosis in the lung cancer xenograft tissues with different treatments. Arrows represented the positive cells.

The effect of miR-26 on NSCLC growth was investigated *in vivo*. The tumor volume was measured in mouse xenografts treated with miR-26 combined with the TGF-β1 inhibitor or JNK inhibitor. The results showed that miR-26 significantly decreased the tumor volume compared with the control (Figure 7A). The protein expression of JNK, LC3 increased following the miR-26 inhibitor treatment, while treatment with the miR-26 inhibitor combined with the TGF-β1 inhibitor or JNK inhibitor reversed the protein expression of JNK and LC3 compared with the miR-26 inhibitor treatment alone (Figure 7B). To determine the role of miR-26 in cell apoptosis *in vivo*, a TUNEL assay was performed on tumor xenograft tissues. The results showed that TUNEL positive cells of the tumor tissue in the miR-26 treatment group, significantly increased in comparison with the control group, while the number of TUNEL positive cells of the tumor tissue remarkably decreased in the groups of the miR-26 combined with the TGF-β1 inhibitor or JNK inhibitor (Figure 7D). In addition, representative results of the immunohistochemical staining of TGF-β1 in the lung cancer tissues are shown in Figure 7C. The results showed that the immunoreactivity of TGF-β1 in the miR-26 inhibitor group decreased in comparison to the control group. Moreover, TGF-β1 intensity significantly attenuated after treatment with the miR-26 inhibitor combined with the TGF-β1 inhibitor in the lung cancer tissues.

## Discussion

This study showed that TGF-β was negatively regulated by miR-26 at the post-transcriptional level in human NSCLC cells. miR-26 induced cell apoptosis and inhibited cell autophagy through the down-regulation of TGF-β in a JNK dependent manner in human NSCLC cells. In addition, miR-26 mimics induced cell apoptosis was associated with ER stress signaling in human NSCLC cells. Down-regulation of ERS led to the inhibition of apoptosis induced by miR-26 mimics.

miR-26 is one of the most significant miRNAs involved in human malignancy. Aberrant expression of miR-26 was found in various types of cancers including esophageal squamous cancer, colorectal cancer and breast cancer (Liu et al., 2015; Li et al., 2017; López-Urrutia et al., 2017). Our study demonstrated that miR-26 expression was lower in NSCLC tissues than in non-tumor tissues. A previous study demonstrated that miR-26 promoted apoptosis of the hepatocellular carcinoma cells through inhibiting autophagy (Johnston et al., 2016). Our study showed that miR-26 mimics induced cell apoptosis in NSCLC cells and promoted caspase-3, 9 activities in NSCLC cells. Interestingly, the miR-26 inhibitor enhanced the protein expression of LC3 and autophagy related genes in NSCLC cells.

TGF-β, a multifunctional cytokine, was involved in various biological processes, including development, cell apoptosis, proliferation, and autophagy through the interaction with several signaling pathways (Katz et al., 2013; Chen et al., 2016; Gratchev, 2017; Ramu et al., 2017). A previous study showed that gramine treatment diminished angiogenesis and induced cell apoptosis by modulating TGF-β signals in hamster buccal pouch (HBP) carcinogenesis (Liu et al., 2016). Many studies have demonstrated that TGF-β induced autophagy in hepatocellular carcinoma cells and mammary carcinoma cells (Sureshbabu et al., 2016; Ren et al., 2017). In this study, we found that miR-26 significantly suppressed TGF-β protein expression and TGF-β activation. TGF-β could down-regulate apolipoprotein M expression through a JNK pathway in the HepG2 cells (Song et al., 2017). In this study, we observed that the miR-26 inhibitor induced autophagy, while the miR-26 induced cell apoptosis by inhibiting TGF-β expression in a JNK-dependent manner. A recent study revealed that the crosstalk of autophagy and apoptosis was involved in the dual role of autophagy under ER Stress (Li et al., 2016). Our results identified that miR-26 mimics enhanced expression of ERS related proteins. Down-regulation of ERS inhibited the apoptosis induced by miR-26 mimics in A549 cells. Interestingly, Down-regulation of ERS failed to affect cell autophagy induced by the miR-26 inhibitor. These results demonstrated that the ERS signaling pathway was associated with miR-26 regulated apoptosis in NSCLC cells.

In conclusion, this study demonstrated that miR-26 induced cell apoptosis and inhibited cell autophagy of NSCLC, through inhibiting TGF-β expression in a JNK dependent manner, both *in vitro* and *in vivo*. Moreover, miR-26 mimics induced cell apoptosis was associated with ER stress in human NSCLC cells. Down-regulation of ERS could reverse the apoptosis induced by miR-26 mimics. Our results provided a novel potential therapeutic target for treatment of NSCLC.

## Author Contributions

YH, HL, and LJ carried out the studies, participated in the experimental design, statistical analysis, and drafted the manuscript. BR participated in the sample collection and statistical analysis. JM and HX conceived the study, participated in its design and coordination, and helped to draft the manuscript. All authors read and approved the final manuscript.

## Conflict of Interest Statement

The authors declare that the research was conducted in the absence of any commercial or financial relationships that could be construed as a potential conflict of interest.
